# The influence of washed microbiota transplantation on menstruation in female patients of childbearing age

**DOI:** 10.3389/fendo.2026.1715020

**Published:** 2026-02-11

**Authors:** Qingting Wu, Mingzhu Wang, Juan Yang, Jiangyan Wang, Shuo Feng, Shenghua Lu, Zhichu Qin, Xingxiang He, Lei Wu

**Affiliations:** 1 Department of Gastroenterology, Research Center for Engineering Techniques of Microbiota-Targeted Therapies of Guangdong Province, The First Affiliated Hospital of Guangdong Pharmaceutical University, Guangzhou, China; 2 Guangdong Provincial Key Laboratory for Research and Evaluation of Pharmaceutical Preparations, Guangzhou, China

**Keywords:** 16S rRNA genes sequencing, gut microbiota, menstrual disorders, quality of life, washed microbiota transplantation

## Abstract

**Background and aims:**

Menstrual disorders are closely related to the disorder of gut microbiota. This study aims to explore the impact of Washed microbiota transplantation (WMT) on the quality of life, depression and anxiety scale scores, and menstrual conditions of female patients of childbearing age.

**Methods:**

The data of female patients of childbearing age who received WMT at the First Affiliated Hospital of Guangdong Pharmaceutical University from February 2023 to February 2025 were collected. A comparative analysis was conducted on the effects of SF-36, SDS, SAS and menstrual conditions in female patients of childbearing age before and after WMT treatment. The changes of gut microbiota before and after WMT were analyzed by 16S rRNA gene sequencing.

**Results:**

A total of 23 female patients of childbearing age were included in this study. WMT significantly improved the scores of GH, SF, MH, RE and VT in SF-36 of female patients of childbearing age and significantly reduced the scores of SDS and SAS (P < 0.05). The MDQ score was negatively correlated with the PF, BP, GH, VT, RE and MH scores in SF-36, and positively correlated with the SDS and SAS scores (P < 0.05). WMT enhanced the α diversity of gut microbiota in female patients of childbearing age, and the Chao1 and Shannon indices were statistically significant (P < 0.05). At the same time, the relative abundance of *Dialister*, *Bifidobacterium*, *Faecalibacterium*, *Roseburia* and *Fusobacterium* increases. The relative abundances of *Bacteroides*, *Agathobacter*, *Prevotella*, *Escherichia-Shigella* and *Ruminococcus* decreased.

**Conclusions:**

WMT treatment can effectively improve the quality of life score of female patients of childbearing age and reduce the scores of depression and anxiety scales. WMT can increase the diversity and abundance of gut microbiota in female patients of childbearing age and improve menstrual conditions, which provides new ideas for future clinical treatment.

## 1 Introduction

Menstruation, as a unique periodic physiological phenomenon of women of childbearing age, indicates that women have entered a mature reproductive state, suggesting that ovarian function is beginning to mature and a large number of follicles in the ovaries are starting to develop. It is a manifestation of female fertility and plays a key role in regulating women’s physiological health and reproduction (1). The regularity of menstruation is mainly controlled by the hypothalamic-pituitary-ovarian axis, which can affect the cycle, menstrual period, menstrual volume, and physical and psychological changes during menstruation. Among them, it is closely related to the quality of life, including disease conditions, emotions, and other aspects (2).

In recent years, the incidence of menstrual disorders among women has been on the rise (3), and related studies have also been increasing year by year. Among them, the multi-faceted relationship between gut microbiota and menstruation can be found (4–6). Some studies have reported that the symbiotic effect of the gut microbiota is related to the regulation of estrogen absorption in the body, thereby influencing the menstrual cycle and dysmenorrhea (6, 7). A microbiota survey of patients with depression and anxiety has found that an imbalance in the gut microbiota can lead to emotional symptoms of premenstrual syndrome through the gut-brain axis (5). Therefore, for the overall health of women and the regulation of reproductive diseases, it is necessary to focus on maintaining the homeostasis of the gut microbiota.

Studies have shown that the gut microbiota constructs a complex “gut-brain-endocrine” regulatory network by metabolizing short-chain fatty acids, neurotransmitter precursors and immunomodulatory factors, significantly influencing the host’s mental health, energy metabolism and reproductive function (8, 9). Washed microbiota transplantation (WMT), as an innovative therapy for reconstructing the gut microbiota, has demonstrated unique advantages in diseases such as inflammatory bowel disease and metabolic syndrome (10, 11). Multiple studies have shown that microbiota transplantation can improve the severity of diseases, course performance and quality of life in patients with various diseases (12, 13). Due to their unique physiological manifestations, female patients of childbearing age have distinctive mechanisms of action on the microbiota and internal environment. This study will conduct further research on female patients of childbearing age to analyze the impact of WMT on quality of life and menstruation. Meanwhile, by combining high-throughput sequencing technology to analyze the characteristics of the gut microbiota, the potential microorganisms that exert effects on the quality of life and menstruation of women of childbearing age through WMT were explored.

## 2 Materials and methods

### 2.1 Research object

Female patients of childbearing age who underwent WMT treatment at the First Affiliated Hospital of Guangdong Pharmaceutical University for various diseases from February 2023 to February 2025. Inclusion criteria: (1) No fecal microbiota transplantation was performed before this treatment; (2) Voluntarily participate in this research survey; (3) Adult females aged 18–45 with the capacity for independent living. Exclusion criteria: (1) The patient has received antibiotic treatment within one month before fecal microbiota transplantation; (2) Lack of ability to live independently. Criteria for withdrawal and termination of the study: (1) The patient voluntarily withdraws from this study due to various factors. Total number of women of childbearing age who received WMT from 2023 to 2025 was 23. Completed the first treatment course (n=23), the second treatment course (n=12), the third treatment course (n=6).

### 2.2 General information collection

This study adopted a prospective design. Data were collected prior to the initiation of each WMT session and at predetermined time points following treatment. General information of female patients of childbearing age who received WMT for the first time from February 2023 to February 2025 was collected, including: name, gender, main diagnosis, age, etc. Collected the MOS item short from health survey (SF-36), Self-Rating Depression Scale (SDS), Self-Rating Anxiety Scale (SAS), and menstrual conditions of patients before the start of each WMT treatment. Subsequently, the questionnaires were collected and analyzed: (1) To analyze the impact of WMT on the scores of the Quality of Life Scale, Self-Rating Depression Scale, and Self-Rating Anxiety Scale of female patients of childbearing age; (2) Analyze the impact of WMT on the menstrual conditions of female patients of childbearing age; (3) Analyze the correlation between the Quality of Life scale and the Self-Rating Depression Scale and the Self-Rating Anxiety Scale; (4) Analyze the changes in the diversity and abundance of intestinal flora in female patients of childbearing age before and after WMT through fecal specimen sequencing; (5) The correlation between gut microbiota and quality of life, depression, anxiety, and menstrual conditions.

### 2.3 Determination of the quality of life scale

the MOS item short from health survey (SF-36) developed by the Medical Outcomes Study (MOS) (14) was adopted, which contains 8 dimensions, namely: physical functioning (PF), role physical (RP), bodily pain (BP), general health (GH), vitality VT), social functioning (SF), role-emotional (RE), mental health (MH). This form is a score for collecting patients’ quality of life through a relatively simple method. It is independently completed by the patient without the influence of external factors.

### 2.4 Determination of the self-rating depression scale

The Self-Rating Depression Scale (SDS) released by William W.K. Zung in 1965 (15) was adopted. This scale is a relatively simple method to collect patients’ perception of their own depression. It is independently completed by the patient without the influence of external factors.

### 2.5 Determination of the self-rating anxiety scale

The Self-Rating Anxiety Scale (SAS) released by William W.K. Zung in 1965 (15) was adopted. This scale is a relatively simple method to collect patients’ perception of their own anxiety situation. It is independently completed by the patient without the influence of external factors.

### 2.6 Determination of the menstrual bleeding scale

The Blood Loss Measured published by Katrina M Wyatt in 2001 (16) was adopted. This form is a relatively simple method for collecting the total amount of bleeding during a patient’s menstrual period. The patient selects the corresponding option based on the bleeding situation during menstruation. Under the condition of no external factors, the patient completes it independently.

### 2.7 Determination of the menstrual symptom scale

The Menstrual Distress Questionnaire (MDQ) released by Moos in 1968 (17) was adopted. This questionnaire is a relatively simple method that assesses the degree of menstrual discomfort in patients using scores. The patient selects the corresponding option based on menstrual symptoms and completes it independently without the influence of external factors.

### 2.8 Donor selection and WMT protocol

This study adopted the standardized preparation process of washing microbiota recommended by the Nanjing Consensus and ensured the safety of microbial transplantation through a multi-dimensional donor screening system. The recruitment scope of donors mainly includes permanent residents and college students in the Guangzhou area. Through structured questionnaire examination, clinical physical examination and laboratory tests (including blood biochemistry, fecal pathogen screening, etc.), candidates with infectious pathogen infection, mental system diseases and other contraindications for transplantation were excluded. The selected donor samples should meet types III-IV of the Bristol Stool Scale, and the single collection volume should be no less than 50g.

In the process of microbiota isolation and purification, the laboratory personnel mixed the fecal samples with sterile physiological saline at a mass-to-volume ratio of 1:5 and used the GenFMTer fully automatic microbial separation system for gradient purification treatment. This system effectively removes large particle residues, parasite eggs and fungal spores and other impurities through multi-stage filtration technology. Subsequently, at room temperature, gradient centrifugation is carried out with a centrifugal force of 1100×g (3 minutes per time), and high-purity microbial precipitate is obtained after repeated washing three times. Finally, the precipitate was resuspended in 100 ml of normal saline to form a standardized microbiota preparation, with its concentration prepared based on the ratio of 1mL of suspension per gram of fecal sample. During the implementation of microbiota transplantation, the appropriate transplantation approach is selected based on the clinical indications of the subjects: for upper gastrointestinal intervention, a nasojejunal TET tube is placed under gastroscopy or directly inserted into the nasojejunal tube, while for lower gastrointestinal intervention, intestinal tube placement is completed through colonoscopy guidance. The treatment cycle is divided into “three three courses”. In the first three months, treatment is conducted once a month. After a three-month interval, consolidation treatment is carried out to promote the stability of microbiota colonization. The total intervention cycle is five months. In each stage, microbiota suspension (120mL/day) is continuously infused through the digestive tract for three consecutive days.

### 2.9 16S rRNA genes sequencing

Genomic DNA extraction from fecal samples was performed using the E.Z.N.A.^®^ Soil DNA Isolation Kit (Omega Bio-tek, USA) in strict accordance with the manufacturer’s protocol. DNA purity and concentration were quantified using a NanoDrop 2000 spectrophotometer (Thermo Fisher Scientific, Wilmington, DE, USA) to establish quality control standards for subsequent analyses. The V3-V4 hypervariable regions of bacterial 16S ribosomal RNA (rRNA) genes were amplified with primers 338F (5′-ACTCCTACGGGAGGCAGCAG-3′) and 806R (5′-GGACTACHVGGGTWTCTAAT-3′), followed by verification of amplicon integrity via 1.5% agarose gel electrophoresis. Purified amplification products were subjected to paired-end sequencing (2 × 300 bp) on the Illumina MiSeq platform by Novogene Bioinformatics Technology Co., Ltd. (Beijing, China).

Raw paired-end sequencing reads were merged using FLASH (v1.2.11) and quality-filtered with FASTP (v0.19.6). Sequences were denoised and chimeras removed via the DADA2 algorithm within the QIIME2 (version 2020.2) pipeline to generate high-resolution amplicon sequence variants (ASVs). To ensure cross-sample comparability, sequences were rarefied to 4,000 reads per sample. Taxonomic annotation was performed against the SILVA 16S rRNA database (v138), followed by microbial community structure analysis using the Novomagic cloud platform (https://magic-plus.novogene.com/).

### 2.10 Statistical analysis

Data analysis was accomplished using SPSS statistical software (version 22.0) developed by IBM Corporation of the United States and GraphPad Prism software (version 8.0.2). The Shapiro-Wilk test was used for the data normality test. The statistical description of quantitative data followed the following rules: data that conformed to a normal distribution were expressed as mean ± standard deviation, while data that did not conform to a normal distribution were described as median (interquartile range). For the inter-group comparison of two paired samples, if both conform to a normal distribution, the paired t-test is used; if there was a non-normal distribution, the Wilcoxon rank sum test was used. If the data conform to the normal distribution, Pearson analysis was used for correlation. If the data do not conform to the normal distribution, Spearman analysis was used for correlation. When the *P* value was less than 0.05, there was a statistical difference. MH, and menstrual cycle showed. Additionally, the False Discovery Rate (FDR) method was applied for correction. A corrected *P* value (i.e., q-value) of less than 0.05 was considered statistically significant.

## 3 Result

### 3.1 The influence of WMT on the quality of life, depression and anxiety scale scores, and menstrual conditions of female patients of childbearing age

**Figure 1:**
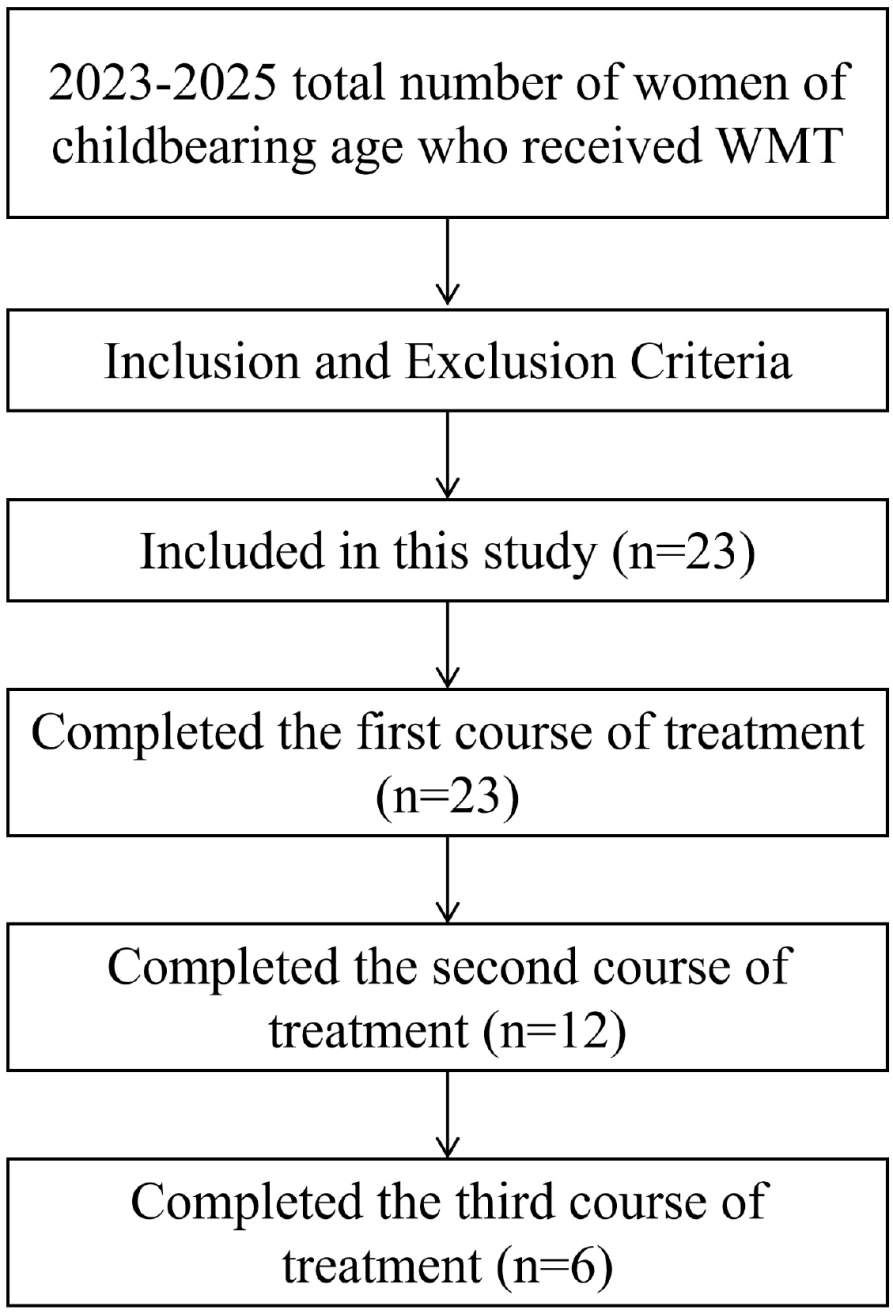
The flowchart illustrating the participant screening process.

A total of 23 female patients of childbearing age completed one course of WMT. A total of 12 patients completed two courses of WMT, and 6 patients completed three courses of WMT (**Figure 1**). Paired tests were used to compare the scores of the quality of life, depression, anxiety, menstrual symptom scale, menstrual cycle, bleeding days and total bleeding volume of female patients of childbearing age after one course of WMT. The research results show that after completing the first course of WMT, it can be found that the quality of life of female patients of childbearing age shows an upward trend in the five dimensions of BP, GH, SF, RE, and MH compared with the baseline, while SDS and SAS show a downward trend, the menstrual cycle shows an increasing trend, and the total amount of bleeding shows a decreasing trend. Among them, SDS (*P* = 0.005), SAS (*P* = 0.044), GH (*P* = 0.001), SF (*P* = 0.008), MH (*P* = 0.001), and menstrual cycle (*P* = 0.012), among which GH, SF, MH, and menstrual cycle showed q < 0.05 and were thus considered statistically significant after FDR correction. The results are shown in **Table 1**.

**Table 1:** Comparison of SF-36, SDS, SAS and menstrual conditions of female patients of childbearing age after one course of WMT.

Indicators	Before WMT	After one course of WMT	*P*	*q*
SDS	53.39 ± 12.72	45.78 ± 18.13	0.005	**0.075**
SAS	47.04 ± 12.12	42.04 ± 19.19	0.044	**0.11**
PF	90.00 (70.00−95.00)	90.00 (75.00−100.00)	0.154	**0.289**
RP	25.00 (0.00−100.00)	25.00 (0.00−100.00)	0.799	**0.999**
BP	60.74 ± 29.34	67.52 ± 28.59	0.165	**0.275**
GH	32.48 ± 19.44	43.65 ± 21.09	0.001	**0.008**
VT	55.65 ± 18.29	55.65 ± 21.67	0.867	**0.929**
SF	64.19 ± 26.19	74.00 ± 26.05	0.008	**0.03**
RE	44.93 ± 43.37	53.62 ± 44.65	0.260	**0.355**
MH	59.13 ± 19.41	65.04 ± 17.07	0.001	**0.005**
Menstrual cycle	28.65 ± 3.90	31.48 ± 4.22	0.012	**0.036**
Bleeding days	5.00 (4.00−7.00)	5.00 (4.00-7.00)	0.865	**0.998**
Total bleeding volume	72.00 (45.00−129.00)	59.00 (48.00−107.00)	0.166	**0.249**
MDQ	45.00 (35.00−66.00)	48.00 (32.00−62.00)	0.099	**0.212**

The False Discovery Rate (FDR) method was applied for correction. In bold: A corrected P value (i.e., q-value) of less than 0.05 was considered statistically significant.

After the completion of the second course of WMT, it can be found that the quality of life of female patients of childbearing age shows an upward trend in the seven dimensions of PF, BP, GH, VT, SF, RE, and MH compared with the baseline, while SDS, SAS, and MDQ show a downward trend. The menstrual cycle shows an increasing trend, and the total amount of bleeding shows a decreasing trend. Among them, SDS (*P* = 0.047), SAS (*P* = 0.014), GH (*P* = 0.004), and RE (*P* = 0.042), but none remained statistically significant after FDR correction (all q > 0.05) The results are shown in **Table 2**.

**Table 2:** Comparison of SF-36, SDS, SAS and menstrual conditions of female patients of childbearing age after second course of WMT.

Indicators	Before WMT	After second course of WMT	*P*	*q*
SDS	54.92 ± 13.24	45.33 ± 15.57	0.047	**0.176**
SAS	49.17 ± 12.51	43.42 ± 13.87	0.014	**0.105**
PF	85.00 (70.00−95.00)	90.00 (80.00−95.00)	0.205	**0.439**
RP	43.75 ± 38.62	43.75 ± 38.62	1.000	**1.154**
BP	57.17 ± 33.60	72.42 ± 27.21	0.224	**0.42**
GH	32.08 ± 18.64	52.08 ± 17.51	0.004	**0.06**
VT	58.33 ± 19.23	60.83 ± 24.39	0.708	**0.885**
SF	67.71 ± 26.36	70.88 ± 27.38	0.596	**0.813**
RE	33.33 (0.00−66.92)	83.33 (8.33−10.00)	0.042	**0.21**
MH	62.67 ± 20.84	84.00 (53.00−92.00)	0.072	**0.216**
Menstrual cycle	29.00 (27.25−31.50)	29.50 (27.25−31.50)	0.438	**0.657**
Bleeding days	5.00 ± 1.54	5.00 ± 1.48	>0.999	**1.070**
Total bleeding volume	75.25 ± 44.51	64.58 ± 32.69	0.248	**0.413**
MDQ	54.42 ± 22.31	45.17 ± 19.00	0.098	**0.245**

The False Discovery Rate (FDR) method was applied for correction. In bold: A corrected P value (i.e., q-value) of less than 0.05 was considered statistically significant.

After completing the third course of WMT, it can be found that the quality of life of female patients of childbearing age shows an upward trend in the seven dimensions of PF, BP, GH, VT, SF, RE, and MH compared with the baseline, while SDS, SAS, and MDQ show a downward trend. The menstrual cycle shows an increasing trend, the number of bleeding days shows an increasing trend, and the total amount of bleeding shows a decreasing trend. Among them, SDS (*P* = 0.008), SAS (*P* = 0.04), VT (*P* = 0.02), and MH (*P* = 0.03), but none remained statistically significant after FDR correction (all q > 0.05). The results are shown in **Table 3**.

**Table 3:** Comparison of SF-36, SDS, SAS and menstrual conditions of female patients of childbearing age after third course of WMT.

Indicators	Before WMT	After third course of WMT	*P*	*q*
SDS	49.67 ± 8.24	37.17 ± 8.57	0.008	**0.12**
SAS	47.50 ± 6.03	38.33 ± 7.53	0.040	**0.15**
PF	75.00 ± 35.21	85.83 ± 16.86	0.197	**0.369**
RP	87.50 (56.25−100.00)	87.50 (0.00−100.00)	0.655	**0.756**
BP	61.00 ± 35.68	74.50 ± 33.88	0.493	**0.672**
GH	39.17 ± 12.42	58.17 ± 18.72	0.070	**0.15**
VT	60.83 ± 25.38	77.50 ± 18.91	0.020	**0.15**
SF	70.83 ± 34.16	85.50 ± 22.84	0.057	**0.171**
RE	61.17 ± 38.98	100.00 ± 0.00	0.059	**0.148**
MH	70.00 (48.00−82.00)	90.00 (83.00−94.00)	0.030	**0.15**
Menstrual cycle	28.17 ± 2.40	28.33 ± 2.58	>0.999	**1.070**
Bleeding days	4.17 ± 1.17	4.00 ± 0.89	0.610	**0.763**
Total bleeding volume	55.55 ± 24.52	46.50 ± 19.88	0.215	**0.358**
MDQ	43.50 ± 15.36	40.33 ± 20.23	0.294	**0.441**

The False Discovery Rate (FDR) method was applied for correction. In bold: A corrected P value (i.e., q-value) of less than 0.05 was considered statistically significant.

### 3.2 The influence of WMT on the α diversity of gut microbiota in female patients of childbearing age

**Figure 2:**
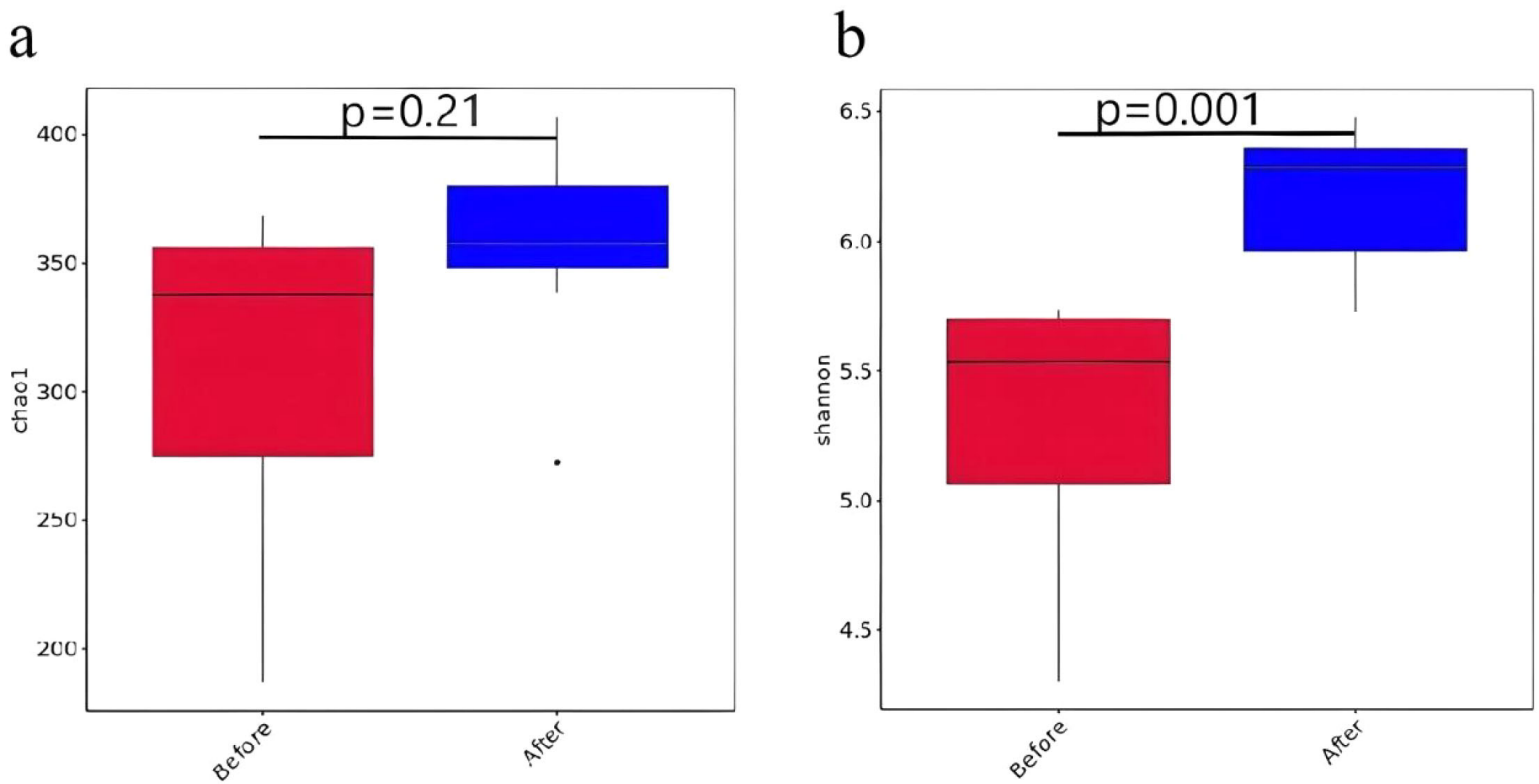
The Chao1 **(a)** and Shannon **(b)** indices of gut microbiota α diversity before and after the first WMT in women of childbearing age.

Inter-group test analysis was conducted on the Chao1 and Shannon indices of female patients of childbearing age before and after one course of WMT treatment. It was found that the chao1 and Shannon indices increased after WMT treatment, among which the Shannon index was statistically significant (*P* < 0.05). Therefore, the diversity of gut microbiota in female patients of childbearing age significantly increased after WMT treatment, and the results are shown in **Figure 2**.

### 3.3 The influence of WMT on the abundance of gut microbiota in female patients of childbearing age

**Figure 3:**
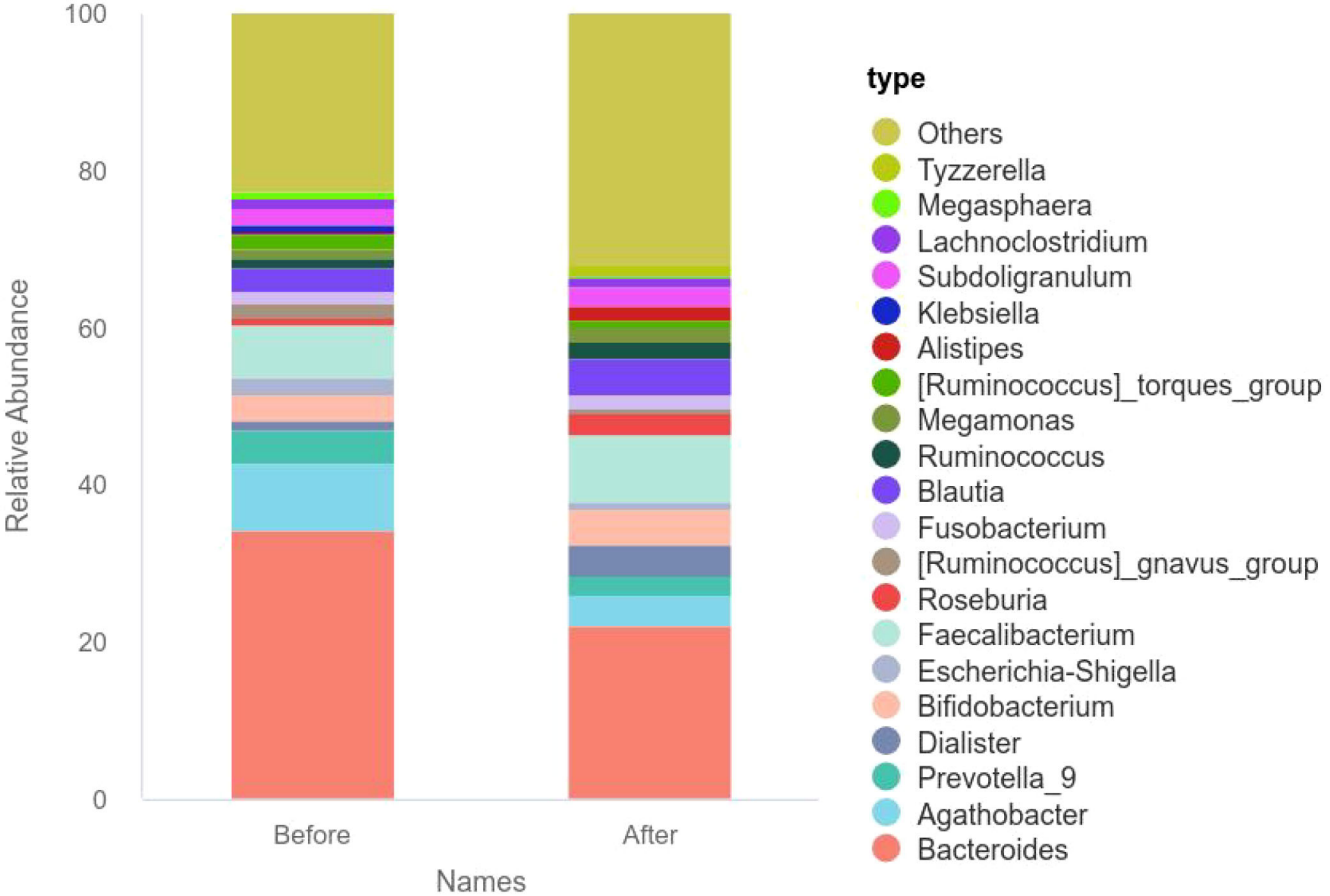
The relative abundance of intestinal microbiota at the genus level in female patients of childbearing age before and after the first WMT session.

The abundance of intestinal flora before and after one treatment course of WMT in female patients of childbearing age was represented by a bar chart. At the genus level, the relative abundances of *Dialister*, *Bifidobacterium*, *Faecalibacterium*, *Roseburia*, and *Fusobacterium* increased after WMT. The relative abundances of *Bacteroides*, *Agathobacter*, *Prevotella*, *Escherichia-Shigella* and *Ruminococcus* decreased. The specific results are shown in **Figure 3**.

### 3.4 The correlation between gut microbiota and the quality of life, depression, anxiety and menstrual conditions of female patients of childbearing age

**Figure 4:**
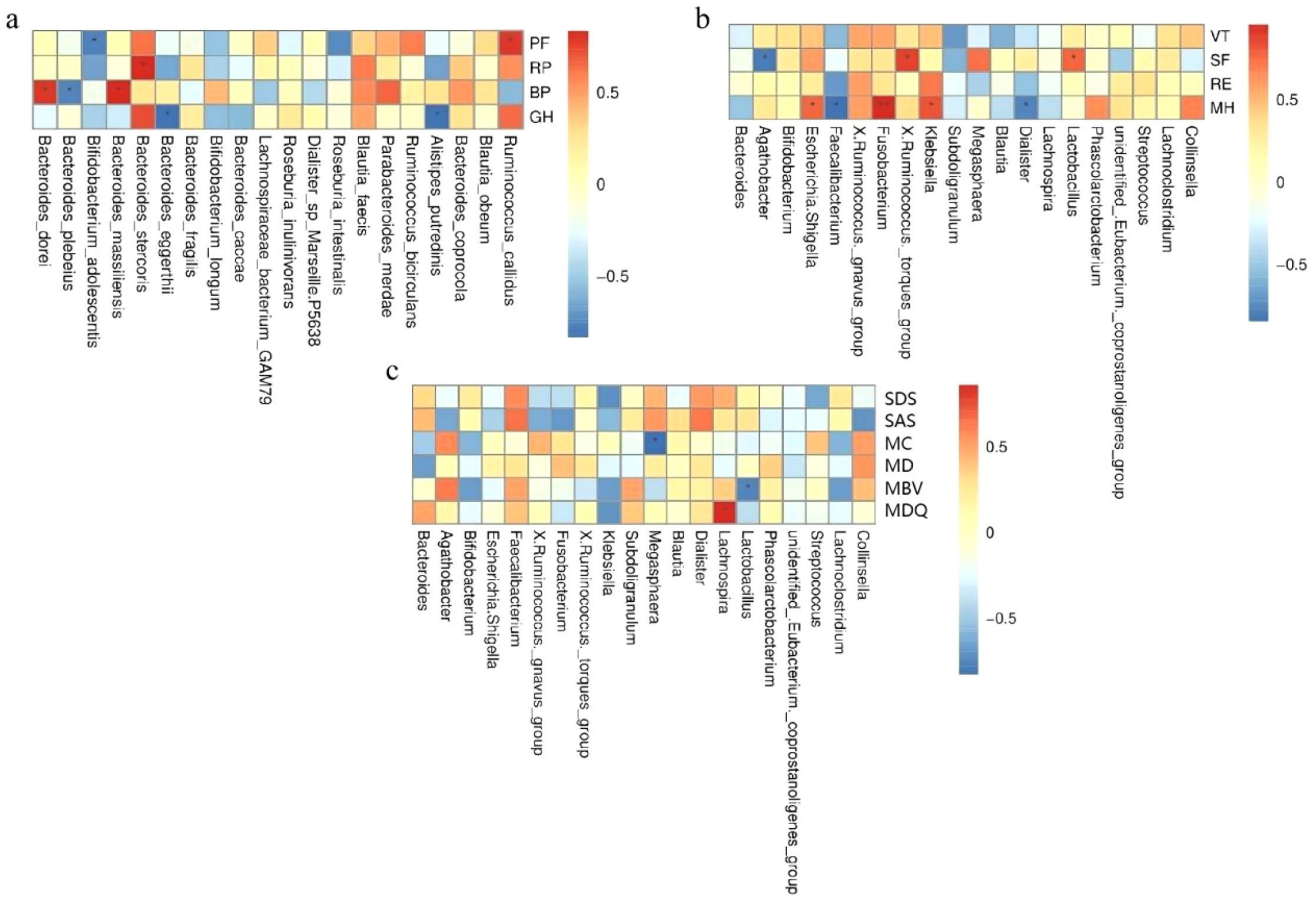
Correlation between gut microbiota and SF-36 **(a, b)**, SDS, SAS, MDQ score, menstrual bleeding volume (MBV), menstrual cycle (MC), and menstrual days (MD) **(c)** in female patients of childbearing age before and after the first WMT session. * indicates *P* < 0.05, and ** indicates *P* < 0.01.

The correlations between each score and gut microbiota were represented using species heatmaps, and the specific results are shown in **Figure 4**. It was found that SF was significantly positively correlated with *Lactobacillus* and *Ruminococcus*, and significantly negatively correlated with *Agathobacter*. MH was significantly positively correlated with *Fusobacterium* and significantly negatively correlated with *Dialister* and *Faecalibacterium*. The menstrual cycle was significantly negatively correlated with *Megasphaera*. The amount of menstrual bleeding is significantly negatively correlated with *Lactobacillus*. MDQ is significantly positively correlated with *Lachnospira*.

## 4 Discussion

This study explored the impact of WMT on the quality of life, depression, anxiety and menstrual conditions of women of childbearing age. The influence of WMT on the SF-36 score of female patients of childbearing age, the influence of WMT on the SDS and SAS scores, as well as the influence of WMT on the menstrual cycle, bleeding days and total bleeding volume were analyzed. The primary outcome measures were the changes in the total SF-36 score and the SDS and SAS scores before and after WMT treatment. Secondary outcome measures included changes in menstrual cycle length, bleeding days, total bleeding volume, MDQ score, as well as alterations in gut microbiota α-diversity and the relative abundance of specific bacterial genera. This study found that WMT significantly improved the quality of life, depression and anxiety of patients of childbearing age, and significantly changed the menstrual cycle. There was a changing trend in the number of bleeding days and the total amount of bleeding, and the menstrual symptoms showed an alleviating trend. Female patients of childbearing age all showed a significant decrease in SDS and SAS scores after three courses of WMT treatment. After the first course of treatment, overall health, social function and mental health in SF-36 could be significantly improved, the menstrual cycle changed significantly, and the total amount of menstrual bleeding showed a decreasing trend. After the second course of treatment, there was a significant improvement in overall health and emotional function in SF-36. Menstrual symptoms showed an alleviating trend, the total amount of menstrual bleeding showed a decreasing trend, and the menstrual cycle showed an increasing trend. After the third course of treatment, the scores of vitality and mental health in SF-36 increased significantly. The menstrual symptoms improved, the total amount of bleeding showed a downward trend, the menstrual cycle showed an increasing trend, and the number of bleeding days showed an increasing trend. This suggests that WMT can effectively improve the quality of life and mental state of patients of childbearing age and may also have a positive impact on menstrual cycles, menstrual flow, menstrual days, and menstrual discomfort symptoms.

According to the mechanism of the hypothalamic-pituitary-ovarian axis, it regulates the changes in the ratio of estrogen and progesterone, resulting in regular endometrial shedding and menstruation. Once the regulation of the hypothalamic-pituitary-ovarian axis is out of control, it will lead to abnormal menstruation and reproductive system diseases. According to research, a large number of women of childbearing age have been troubled by menstrual-related diseases (3). 90% of women of childbearing age experience retrograde menstruation, which can lead to the occurrence of endometriosis. Its main symptom is chronic pelvic pain, especially during menstruation, it can cause dysmenorrhea, which is closely related to the changes in the structure of the gut microbiota (7). The reproductive system related to female menstruation is regulated in a coordinated manner by the endocrine system. If it is disrupted, it may lead to various diseases. The gut microbiota is regarded as an extended endocrine organ and is an important regulatory factor for female reproductive health and related diseases (18). Although this study did not directly measure hormone levels, previous research has demonstrated that the gut microbiota can influence host hormone levels, including estrogen levels in women (6), and plays a significant role in female reproductive activities and overall health. Therefore, we speculate that WMT may affect the menstrual cycle through similar pathways, though this requires future studies to directly measure hormonal changes for confirmation.

This study, by exploring the impact of WMT on the menstrual cycle and accompanying symptoms of women of childbearing age, provides important evidence-based medical evidence for the application of targeted intervention of gut microbiota in the treatment of gynecological diseases. The results of this study show that WMT significantly alters the menstrual cycle by reconstructing the gut microbiota. The number of bleeding days and the total amount of bleeding show a changing trend, and menstrual symptoms show an alleviating trend. This is in line with the research that the gut microbiota regulates the levels of short-chain fatty acids (such as butyrate), improves the microenvironment of endometrial inflammation, and alters the ratio of estrogen and progesterone in women, thereby optimizing the regulation of the menstrual cycle (18). At the same time, this study also found that the improvement of the quality of life score of patients after WMT treatment was significantly correlated with the improvement of menstruation. Combined with the research results (19, 20), exercise can improve physical fitness, relieve physical stress, improve negative emotions, menstrual cycle and fertility. It is recommended that women regularly engage in physical activities to improve their physical health and quality of life.

In terms of emotion regulation, the results of this study are consistent with the classic theories of the gut-brain axis in a large number of studies. Research indicates that the gut microbiota affects the central nervous system through metabolic substances, thereby causing the occurrence of diseases, including depression and anxiety (21). In this study, the depression and anxiety scores showed a significant decrease after WMT treatment, improving the negative emotions of the patients. Multiple studies have shown that fecal microbiota transplantation can effectively improve patients’ depression and anxiety and plays a key role in the prevention and control of mental disorders (22, 23). At the same time, this study found a positive correlation between the menstrual symptom scale and the self-rating scales of depression and anxiety, indicating that emotions have a crucial impact on neuroendocrine and play a certain role in reproductive function. Studies have shown that patients’ acquisition of positive emotions plays a regulatory role in reducing pain perception (24, 25), which suggests that reproductive health and mental health have a synergistic effect in WMT treatment.

This study analyzed the alpha diversity index of female patients of childbearing age before and after one course of WMT treatment and found that the Shannon index of the gut microbiota before and after WMT was statistically significant. After the discovery of WMT at the genus level, the relative abundance of *Dialister*, *Bifidobacterium*, *Faecalibacterium*, *Roseburia* and *Fusobacterium* increased. The relative abundances of *Bacteroides*, *Agathobacter*, *Prevotella*, *Escherichia-Shigella* and *Ruminococcus* decreased. The diversity and abundance of gut microbiota are related to human health. High α -diversity microbiota inhibit the colonization of opportunistic pathogenic bacteria through spatial occupation effects and nutrient competition. For instance, the synergistic effect of *Bacillota* and *Bacteroidota* can reduce the adhesion of *Salmonella* (26, 27). Meanwhile, the metabolic products of the microbiota, such as short-chain fatty acids (SCFAs), promote the growth and proliferation of probiotics by lowering the pH value of the intestinal tract, inhibit the growth of pathogenic and foreign bacteria, and increase the colonization resistance of the intestinal tract, thereby altering the structure of the gut microbiota (26, 28). When α -diversity decreases, the reduction of butyrate, a metabolic product of the microbiota, can lead to the down-regulation of tight junction protein expression in intestinal epithelial cells, thereby disrupting the integrity of the intestinal physical barrier (26). This barrier dysfunction promotes the entry of lipopolysaccharide (LPS) into the bloodstream, activates the NF-κB pathway and triggers chronic inflammatory responses (26). α -diversity maintains immune homeostasis by regulating the Th1/Th2 balance. Research has found that a decrease in diversity is associated with an increase in IL-17 secretion, and this pro-inflammatory state is an important inducement for the occurrence of inflammatory bowel disease (IBD) (29). In addition, butyric acid-producing bacteria inhibit excessive immune responses by inducing the differentiation of regulatory T cells (30). The gut microbiota affects neurotransmitter synthesis through the gut-brain axis. A decrease in α -diversity can lead to abnormal metabolism of tryptophan, the precursor of 5-hydroxytryptamine (5-HT), and the deficiency of 5-HT levels usually causes melancholic behaviors (31). α -diversity affects the efficiency of carbohydrate fermentation. When diversity decreases, the reduced production of SCFAs can lead to insufficient energy supply to the intestinal mucosa (32). Therefore, the intrinsic mechanism of the effects of α -diversity of the gut microbiota on physiology and pathology can serve as the basis for maintaining human health and diagnosing and treating diseases. Based on the results of this study, it can be concluded that WMT treatment can increase the α diversity of gut microbiota in female patients of childbearing age and improve their quality of life.

It should be noted that this study focuses on bacterial communities. The gut microbiota also encompasses archaea, viruses, fungi, and their metabolites, which interact with bacteria to collectively maintain host health. For example, the gut virome can indirectly influence host metabolism and immune function by modulating bacterial community structure. Future research incorporating metagenomic or metatranscriptomic analyses would contribute to a more comprehensive understanding of the impact of WMT on the overall gut microbiota and its functions.

The dominance of *Lactobacillus* in the cervical and vaginal microbiome among women of childbearing age is a characteristic of cervical and vaginal health. Among them, lactic acid bacteria can keep the cervix and vagina environment acidic, which maintains a low pH value and low inflammation in the cervix and vagina microenvironment, which is crucial for the health of the cervix and vagina (33). The reduction of gut microbiota diversity leads to local and systemic inflammation by up-regulating pro-inflammatory cytokines, while the increase of pro-inflammatory cytokines and immune cells results in the imbalance of the mucosal flora of the cervix and vagina. At the same time, the activity of β -glucuronidase in the intestinal tract increases, raising circulating estrogen. The increase in estrogen can overstimulate the endometrial tissue, leading to infertility, irregular menstrual cycles, or causing uterine inflammation (34–36). Studies on mice have found that the diversity of gut microbiota associated with endometriosis is reduced (37). At the genus level, Huang et al. (34) reported an increase in the populations of harmful anaerobic bacteria (*Eggerthella lenta*) and *Eubacterium dolichum* in the intestines of women with endometriosis. The populations of other microorganisms decreased significantly, among which the abundances of *Clostridium botulinum*, *Ruminococcus* and *Lachnospira* were reduced. These microbial symbionts produce short-chain fatty acids that regulate intestinal integrity and are associated with various other diseases related to gut microbiota imbalance, such as Crohn’s disease (34). Among patients with gastrointestinal symptoms, *Bacteroidota* is associated with constipation, abdominal distension, flatulence, vomiting and nausea (35). The abundance of *Lactobacillus*, *Ruminococcus* and *Fusobacterium* in letrozole-induced Polycystic Ovary Syndrome (PCOS) rats was lower, while the abundance of *Prevotella* was higher than that in the control rats (38). After treatment with lactic acid bacteria, the estrous cycle of 75% of rats was improved, indicating that a single genus may have a profound impact on the estrous cycle of PCOS patients. It was also found that the intestinal microbiota recovered, with increased levels of lactic acid bacteria and *Fusobacterium* and decreased levels of *Prevotella* (38). In addition, lactic acid bacteria can significantly improve mood and reduce stress by maintaining the balance of gut microbiota. Lactic acid bacteria improve mood and cognitive function by producing neurotransmitters such as serotonin, dopamine and gamma-aminobutyric acid (GABA) (39, 40). Meanwhile, a study found that lactic acid bacteria can effectively prevent and treat mental health problems by regulating the gut microbiota and the immune system (41). The alteration of gut microbiota abundance plays a significant role in human physiology and pathology, which is consistent with the result in this study that while WMT was used to treat life diseases in female patients of childbearing age, their quality of life also improved. In this study, we further explored the influence of WMT on menstruation in female patients of childbearing age, laying the foundation for subsequent research on the effects of environmental factors (42), gut microbiota (43–45), metabolic biomarkers (46, 47), and the influence of WMT (48–54) on diseases.

This study has several limitations. First, the small sample size (n=23) and the attrition of participants over the treatment courses may reduce statistical power and limit the generalizability of the findings. Future studies with larger sample sizes and multi-center designs are needed to validate these results. Second, the outcome assessments primarily relied on patient self-reported scales, which are subject to subjective bias. Although standardized scales were used, future research should incorporate objective clinical indicators, such as hormone levels and inflammatory markers. Third, this study did not systematically control for or analyze potential confounding factors that could influence menstruation, such as underlying diseases (e.g., inflammatory bowel disease, irritable bowel syndrome), concomitant medications (e.g., probiotics), or lifestyle factors. In particular, supplementation with probiotics (e.g., Lactobacillus spp.) may alter the proportion of *Bacillota*, potentially introducing bias. Fourth, as an observational study, this research cannot establish causality. The specific molecular pathways through which WMT influences menstruation (e.g., the microbiota-gut-brain-gonadal axis) require further elucidation through animal experiments or mechanistic studies. Finally, the correlation heat map in the article is a preliminary exploratory analysis. Its core purpose is to visualize the association trend between the microbiota and clinical variables/intervention groups and generate research hypotheses. Therefore, no multiple comparison correction or confounding factor control was conducted in the initial stage. Further verification through confirmatory analysis is required subsequently.

This study systematically reveals the improvement effect of WMT on the quality of life and emotional state of women of childbearing age, as well as the intrinsic connection of possible mutual influence with menstrual health by integrating modern microecological theory with clinical practice. The increase in the diversity and abundance of gut microbiota in this study can improve the pathological state and health level of female patients of childbearing age, thereby enhancing their quality of life. At the same time, it indirectly verifies the association between the quality of life and some genera in this study.

## 5 Conclusions

WMT treatment can effectively improve the quality of life score of female patients of childbearing age and reduce the scores of depression and anxiety scales. WMT can increase the diversity and abundance of gut microbiota in female patients of childbearing age, improve menstrual conditions, and provide innovative ideas for future clinical treatment.

## Data Availability

The authors declare that all the data and the material used in this study are available within this article. The sequence data for all samples have been deposited in the NCBI Sequence Read Archive (SRA) under accession code BIOProject: PRJNA1404473. All data generated or analysed during this study are available from the corresponding authors upon reasonable request.
